# Solvation Thermodynamics and Its Applications

**DOI:** 10.3390/e26020174

**Published:** 2024-02-18

**Authors:** Arieh Ben-Naim

**Affiliations:** Department of Physical Chemistry, The Hebrew University of Jerusalem, Edmond J. Safra Campus, Givat Ram, Jerusalem 91904, Israel; arieh.ben-naim@mail.huji.ac.il

**Keywords:** solvation, thermodynamics, Gibbs energy of solvation

## Abstract

In this article, we start by describing a few “definitions” of the solvation processes, which were used in the literature until about 1980. Then, we choose one of these definitions and show that it has a simple molecular interpretation. This fact led to a *new definition* of the *solvation process* and the corresponding thermodynamic quantities. The new measure of the solvation Gibbs energy has a simple interpretation. In addition, the thermodynamic quantities associated with the new solvation process have several other advantages over the older measures. These will be discussed briefly in the third section. In the fourth section, we discuss a few applications of the new solvation process.

## 1. Introduction

In this article, we narrate the development story of a new measure of solvation that occurred in the late 1970s, which was summarized in the book “Solvation Thermodynamics” in 1987 [1]. The term “solvation thermodynamics” was originally used within the context of the study of aqueous solutions of electrolyte solutions, Gurney (1953) [2]. In that context, the term “hydration thermodynamics” was more commonly used. In fact, the term “solvation” was not properly defined before 1978. In the Encyclopedia Britannica [3], we find the following definition of this term:

*“When a solvent and a solute molecule link together with weak bonds, the process is called solvation”.*

In Gurney’s classical book (1953) [2], we find the following “definition” of solvation:

*“The interaction that takes place when an ion is introduced into a solvent is called the solvation of the ion”.*

Neither of these may be considered a proper definition of the solvation process. Note also that in both “definitions”, there is a distinction between a “solute” and a “solvent”. In fact, such a distinction was essential in the older approach to solvation thermodynamics. This was removed in the new definition of the “*solvation process*”.

In practice, most studies of solvation thermodynamics started with the well-known expression for the chemical potential (CP) of a solute *s*, in a solvent *l* (usually water, *w*), in the limit of very dilute solution [1,4]:

(1) $$\mu_{S}=\mu_{S}^{O}+RT\ln C_{S}$$

where *R* is the gas constant, *T* is the absolute temperature, and $C_{S}$ is some measure of the concentration of *s* in the solution. The quantity $\mu_{S}^{O}$ is referred to as the *standard* CP.

It should be emphasized that Equation (1) is valid only for very dilute solution of *s* in the solvent. The concentration $C_{S}$ can be either the *mole fraction*, the *molarity*, or the *molality* of *s* in the solution. The definition of these and the relationship between them are provided in reference [1].

Based on Equation (1), there were at least three different processes of solvation. The process can be symbolically written as:

(2) $$\left[\begin{array}{c} g\to l\\ C_{s}^{g}=C_{s}^{l} \end{array}\right]$$

This means the transfer of one solute *s* (or one mole of *s*) from an ideal-gas phase to a dilute solution of *s* in the liquid *l*, in which $C_{s}^{g}=C_{s}^{l}$. The Gibbs energy change for this process may be written as:

(3) $$∆G\left[\begin{array}{c} g\to l\\ C_{s}^{g}=C_{s}^{l} \end{array}\right]=\mu_{S}^{l}-\mu_{S}^{g}=\mu_{S}^{Ol}-\mu_{S}^{Og}+RT\ln{\left(C_{s}^{l}/C_{s}^{g}\right)}_{C_{s}^{g}=C_{s}^{l}}=\mu_{S}^{Ol}-\mu_{S}^{Og}$$

The quantity $\mu_{S}^{Ol}-\mu_{S}^{Og}$ is referred to as the standard Gibbs energy of solvation (in the old literature, it was referred to as “free energy of solution”) based on the specific concentration scale $C_{S}$. The value of $\mu_{S}^{Ol}-\mu_{S}^{Og}$ can be determined by the condition of equilibrium:

(4) $$\mu_{S}^{l}=\mu_{S}^{g}$$

from which we obtain:

(5) $$\mu_{S}^{Ol}-\mu_{S}^{Og}=-RT\ln{\left(C_{s}^{l}/C_{s}^{g}\right)}_{eq}$$

where the ratio on the right-hand side of Equation (5) is evaluated at equilibrium between the two phases.

At this stage, we have at least three different standard Gibbs energies of transfer, denoted ${∆\mu }^{o}\left(g\to l\right)$. The literature is full of arguments regarding the advantages of one standard quantity over another. Based on thermodynamics alone, one could not decide on which standard quantity should be preferred. In fact, it is not clear which of these quantities is truly a measure of the “average interaction” between the solute and the solvent. For instance, Tanford (1973) [5] wrote:

*“For reasons first given by Gurney (1953) and reiterated by Kauzmann (1959 [6]), free energies of transfer from one solvent to another should be expressed in units, which simply means that the solute concentration in the equation for the chemical potential is expressed in mol fraction units”.*

This statement was criticized by Ben-Naim (1978) [7]. Specifically, it was claimed that Tanford’s statement about Gurney’s and Kauzmann’s “*reasons*” for using mole fractions was not true and misleading. Indeed, both Gurney [2] and Kauzmann [6] *did use* the mole fraction, in the standard CP, but did so simply because it was quite a common practice at that time. They never gave *reasons* as to why “*free energy of transfer from one solvent to another should be expressed in unitary units*”.

As a reaction to my criticism, Tanford (1979) [8] sent a letter to the editor, admitting that he did not understand my criticism, yet insisting on the usage of mole fractions in the definition of the standard free energy of solvation. 

To conclude this section, let us say that in 1978, it was shown that the *standard* free energy of transferring a solute from *g* to *l* based on the *molarity* scale has a simple molecular interpretation. It is the Gibbs energy change for transferring a solute from a fixed position in an ideal gas phase to a fixed position in the liquid. The statistical mechanical expression for this Gibbs energy change was denoted ${∆\mu }^{*}$ (see Section 2) and for simple solutes is equal to:

(6) $${∆\mu }_{s}^{*}\left(g\to l\right)=-kT\ln{\langle \exp\left[-\beta B_{s}\right]\rangle }_{o}$$

Here, *k* is the Boltzmann constant, when we use ${∆\mu }_{s}^{*}$ per molecule. This should be replaced by the gas constant *R*, when we transfer one mole of solute molecules. $B_{s}$ is the total interaction energy of the solute *s* to all other particles in the liquid, ${\beta =\left(kT\right)}^{-1}$, and the average denoted ${\langle \;\rangle }_{o}$ is over all configurations of the solvent molecules using the distribution of solvent configurations *before* the addition of the solute at a fixed position.

## 2. The New Definition of the Solvation Process and the Corresponding Thermodynamic Quantities

In this section, we introduce the new process of *solvation*. Unlike the conventional standard processes of solvation, we start with a molecular process; hence, statistical mechanical considerations apply to this process. Once the solvation process is defined, one can proceed to define all the thermodynamic quantities associated with this process.

We start with the thermodynamic definition of the chemical potential (CP). It is well known that there are various possible definitions of the CP, and the most common and useful ones are:

(7) $${\mu =\left(\frac{\partial A}{\partial N}\right)}_{T,V}={\left(\frac{\partial G}{\partial N}\right)}_{T,P}$$

The first is the derivative of the Helmholtz energy (*A*) with respect to *N* at constant *T* and *V*. The second is the derivative of the Gibbs energy (*G*) with respect to *N* at constant *T* and *P*. 

Next, we derive the statistical mechanical expression for the CP for a one-component system described thermodynamically by the parameters $\left(T,V,N\right)$. We assume that the total interaction energy among the *N* particles is *pairwise additive*. This means that we can write the total potential energy $U_{N}\left(R_{1},\ldots {,R}_{N}\right)$ as:

(8) $$U_{N}\left(R_{1},\ldots ,R_{N}\right)=\sum_{i\neq j}^{\;}U_{N}\left(R_{i},R_{j}\right)$$

where the sum on the right-hand side of Equation (8) is over all the different pairs of particles (i≠j). We also assume that the potential energy of the interaction depends on the locations $R_{1}$ and $R_{j}$. Following this assumption, we use the classical partition function of the system to obtain a general expression for the CP in a T,V,N system.

(9) $$\begin{array}{c} \mu =A\left(T,V,N+1\right)-A\left(T,V,N\right)\\ \;=-kT\ln\left[\frac{Q\left(T,V,N+1\right)}{Q\left(T,V,N\right)}\right]\\ \;=-kT\ln\left[\frac{q^{N+1}\Lambda^{3N}\left(N!\right)Z_{N+1}}{\Lambda^{3\left(N+1\right)}\left(N+1\right)!q^{N}Z_{N}}\right] \end{array}$$

$Q\left(T,V,N\right)$ is the partition function of a system characterized by $\left(T,V,N\right)$, *q* is the internal partition function, and $Z_{N}$ is the configurational partition function defined by:

(10) $$Z_{N}=\int \cdots \int {dR}_{1}\cdots {dR}_{N}\exp\left[-{\beta U}_{N}\right]$$

(11) $$Z_{N+1}=\int \cdots \int {dR}_{0}{dR}_{1}\cdots {dR}_{N}\exp\left[-{\beta U}_{N+1}\right]$$

The locational coordinate of the added particle to the system is denoted by $R_{0}$, and the integrations are over the entire volume of the system *V.* Note that in Equations (10) and (12), we consider a system of spherical particles. The generalization to non-spherical particles is quite straightforward [1].

We now assume that the total potential energy of interaction in the system may be written as:

(12) $$U_{N+1}\left(R_{0},\ldots ,R_{N}\right)=U_{N}\left(R_{1},\ldots ,R_{N}\right)+\sum_{i=1}^{N}U\left(R_{0},R_{i}\right)=U_{N}\left(R_{1},\ldots ,R_{N}\right)+B_{O}$$

where $B_{O}$ is the total interaction energy of the newly added particle at $R_{0}$, with all other particles in the system being at a specific configuration $R_{1},\ldots ,R_{N}$. This quantity is referred to as the *binding energy* of the added particle to the rest of the system.

Next, we write the expression for the CP as:

(13) $$\exp\left[-\beta \mu \right]=\frac{q}{\Lambda^{3}\left(N+1\right)}\int \cdots \int dR_{0}dR_{1}\cdots {dR}_{N}\mathrm{Pr}\left(R^{N}\right)\exp\left[-\beta B_{0}\right]$$

Here, $\mathrm{Pr}\left(R^{N}\right)$ is the probability density of finding the *N* particles in the specific configuration $R_{1},\ldots ,R_{N}$, which is defined by:

(14) $$\mathrm{Pr}\left(R^{N}\right)=\mathrm{Pr}\left(R_{1},\ldots ,R_{N}\right)=\frac{\exp\left[-\beta U_{N}\right]}{\int \cdots \int dR_{1}\cdots {dR}_{N}exp\left[-\beta U_{N}\right]}$$

We can simplify Equation (13) by transforming to relative coordinates $R_{i}^{\prime }=R_{i}-R_{0}$ and noting that $B_{0}$ depends only on the relative coordinates (not the “absolute” ones $R_{i}$). Hence, we can rewrite Equation (13) as:

(15) $$\begin{array}{c} \exp\left[-\beta \mu \right]=\frac{q}{\Lambda^{3}\left(N+1\right)}\int {dR}_{0}\int dR_{i}^{\prime }\cdots dR_{N}^{\prime }\mathrm{Pr}\left(R_{1}^{\prime },\ldots R_{N}^{\prime }\right)\exp\left[-\beta B_{0}\right]\\ \;\;\;\;=\frac{qV}{\Lambda^{3}\left(N+1\right)}{\langle \exp\left[-\beta B_{0}\right]\rangle }_{T,V,N} \end{array}$$

In Equation (15), the inner integrations are over the coordinates $R_{1}^{\prime },\ldots ,R_{N}^{\prime }$. Since this integral is independent of $R_{0}$, we can integrate over $R_{0}$ and obtain the volume *V*. Also, since *N* is very large, the quantity $\left(N+1\right)/\approx N/V=\rho$ is the number density of the particles in the system. The notation ${\langle \;\rangle }_{T,V,N}$ means an average over all the coordinates of the *N* particles with the probability distribution *before* we added the new particle in the system.

The final expression for the chemical potential for this system is:

(16) $$\mu =kT\ln\left[{\rho \Lambda }^{3}q^{-1}\right]-kT\ln{\langle \exp\left[-\beta B_{0}\right]\rangle }_{T,V,N}$$

We derived Equation (16) for the case of a one-component system of simple particles, i.e., the interaction between each pair depends only on the locations of the two particles. The generalization for a mixture of any number of components is straightforward. The CP of a specific species, say, α, in any solvent is:

(17) $$\mu_{\alpha }=kT\ln\left[\rho_{\alpha }\Lambda_{\alpha }^{3}q_{\alpha }^{-1}\right]-kT\ln{\langle \exp\left[-\beta B_{\alpha }\right]\rangle }_{T,V,N}$$

Here, $\rho_{\alpha }\Lambda_{\alpha }^{3}$ and $q_{\alpha }$ have the same meaning as in Equation (16) but for the species α. $B_{\alpha }$ is the binding energy of a single added α-particle to a system with a composition $N=N_{1},N_{2},\ldots ,N_{c}$, and $N_{j}$ is the number of particles of the *j*th species in the system. Note again that the average in Equation (17) is over all the coordinates of all the particles in the system *before* we added the new particle of species α.

That mentioned above is valid for *rigid* non-spherical particles. By “rigid”, we mean that the we can neglect any change in the internal rotational degrees of freedom as well as vibrations about the chemical bonds, when the solute is transferred from the gas into the liquid. In more complicated cases, such as proteins, there are also internal rotational degrees of freedom. This case requires special treatment, which we will not discuss here.

Next, we derive an expression for the pseudo-chemical potential. As for the CP, we first define the pseudo-chemical potential for a one-component system consisting of simple particles. 

Similarly, we define the pseudo-chemical potential by:

(18) $$\mu^{*}=A\left(T,V,N+1;R_{0}\right)-A\left(T,V,N\right)$$

Thus, instead of the process of adding one particle to the system at $\left(T,V,N\right)$, in Equation (9), we add the new particle to a specific location, denoted $R_{0}$ in the system $\left(T,V,N\right)$. 

The statistical mechanical expression for $\mu^{*}$ is similar to Equation (15), with a few differences, i.e.,

(19) $$\begin{array}{c} \mu^{*}=A\left(T,V,N+1;R_{0}\right)-A\left(T,V,N\right)\\ =-kT\ln\left[\frac{Q\left(T,V,N+1;R_{0}\right)}{Q\left(T,V,N\right)}\right]\\ =-kT\ln\left[\frac{q^{N+1}\Lambda^{3N}N!}{q^{N}\Lambda^{3N}N!}\frac{\int \cdots \int dR_{1}\cdots dR_{N}\exp\left[-\beta U_{N+1}\left(R_{0},\ldots ,R_{N}\right)\right]}{\int \cdots \int dR_{1}\cdots dR_{N}\exp\left[-\beta U_{N}\left(R_{1},\ldots ,R_{N}\right)\right]}\right] \end{array}$$

Note that when we add a single particle to a system $\left(T,V,N\right)$ at a fixed position, we also add the *internal* partition function of that particle. However, the newly added particle does not carry a momentum partition function. Also, in Equation (9), we had *N* and N+1 indistinguishable particles in the initial and final states, but in Equation (19), we have *N* indistinguishable particles in both states. The reason is that the added particle to a fixed position is distinguishable from the *N* particles of the same species. Furthermore, since the added particle to a fixed location is devoid of translational degrees of freedom, the two integrals in Equation (19) are on all the locations of the *N* particles.

Next, we can follow the same mathematical steps as we did for the CP and convert Equation (19) to a simple form as:

(20) $$\mu^{*}=kT\ln q^{-1}-kT\ln{\langle \exp\left[-\beta B_{0}\right]\rangle }_{T,V,N}$$

where the average in (20) has the same meaning as in Equation (15).

We next combine the Equation for the CP and the pseudo CP to obtain:

(21) $${\mu =\mu }^{*}+kT\ln\left({\rho \Lambda }^{3}\right)$$

Equation (21) together with interpretation of $\mu^{*}$ in Equation (20) are the most important ones in the study of solvation thermodynamics [9].

**Figure 1:**
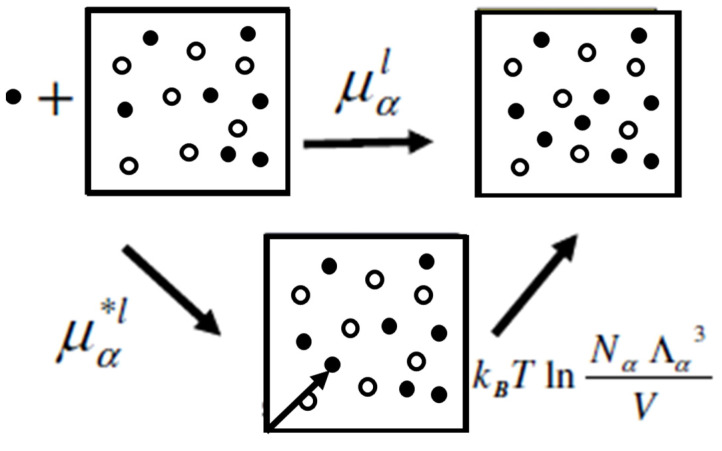
The process of adding a new particle *α* (black particle) into the system (of black and white particles) is split into two steps: first, the particle is placed at a fixed position, then it is released. The corresponding changes in Gibbs energies are the pseudo CP and the liberation Gibbs energy.

Recall that the chemical potential is the change in the Helmholtz or Gibbs energy for adding one particle to a system characterized by the variables T,V,N or $\left(T,P,N\right)$. The two terms in Equation (21) correspond to performing the same process of adding a new particle, in two steps; first, we add the new particle to a fixed position $R_{0}$, and second, we release the particle from the fixed position. Clearly, due to the molecule collisions, the new particle will start to move, wander in the entire volume *V*, and will acquire a distribution of momenta as all the other *N* particles in the system at equilibrium. The corresponding changes in the Helmholtz (or Gibbs) energy are $\mu^{*}$ and $kT\ln\left({\rho \Lambda }^{3}\right)$. The latter is referred to as the “liberation” Helmholtz (or Gibbs) energy. This two-step process is shown in Figure 1. In Ben-Naim (1987 [1]), it was suggested to refer to the newly added molecule of species α as the “solvaton”.

It is important to remember the “molecular content” of the two terms on the right-hand side of Equation (21).

The pseudo-chemical potential has two factors: one contains the internal partition function (*q*) of a single molecule, and the second, an average of the quantity $\exp\left[-\beta B_{0}\right]$, which is a measure of the strength of the interaction energy of the added particle with the rest of the system. This was sometimes referred to as the “free energy of interaction”.

The simplest example of a thoroughly studied solvation Gibbs energy is the case of a hard sphere, and the Helmholtz (or Gibbs) energy associated with its solvation may be calculated by the scaled-particle theory (SPT) [10,11,12,13,14]. This quantity is also called the work associated with creating a cavity of a suitable size in the system. For details, see Ben-Naim (2006) [9]. The work for creating a cavity is used also as a first step in the calculation of the Gibbs energy of solvation for real simple solute such as argon or methane [1,15,16,17]. It should be added that one of the most fascinating problems in solvation thermodynamics was the question regarding the anomalous large and negative entropy of solvation of gases in water [1,18]. This problem and related problems associated with solvation thermodynamics were discussed by many authors [1,9,19,20,21,22].

The liberation term (i.e., the second term) on the right-hand side of Equation (21) is independent of the interaction between the added particle and the rest of the system. It contains three different contributions. First, the particle at the fixed position is devoid of momentum. Once it is released, it acquires a distribution of momenta, hence, the momentum partition function $\Lambda^{3}$. Second, the released particle from the fixed location $R_{0}$ can now access the entire volume of the system *V* (in the T,P,N ensemble, *V* is replaced by the average volume $\bar{V}$). The corresponding contribution to the liberation term is −kTln⁡V. Finally, the particle at $R_{0}$ is of the same species as the other *N* particles in the system. However, being at a fixed position makes it indistinguishable from all the other particles in the system. When this particle is released, it becomes indistinguishable from the other particles of the same species. This process is called *assimilation* and was introduced in the study of the so-called “entropy of mixing”, see Ben-Naim (2006) [9]. The contribution of the assimilation process to the liberation Helmholtz energy is kTln⁡N. Together, the three factors combine to form the dimensionless quantity ${\rho \Lambda }^{3}$ under the logarithm sign.

In the limit of ideal dilute solution, Equation (21) will have the same form, except that now, the pseudo-chemical potential becomes independent of the density of the relevant species, say, α, i.e.,

(22) $$\mu_{\alpha }=\mu_{\alpha }^{*}+kT\ln\rho_{\alpha }\Lambda_{\alpha }^{3}\to \left[\mu_{\alpha }^{*0}+kT\ln\Lambda_{\alpha }^{3}\right]+kT\ln\rho_{\alpha }{=\mu }_{\alpha }^{0}+kT\ln\rho_{\alpha }$$

This is the reason as to why we view the pseudo-chemical potential as the generalized “standard” chemical potential in an ideal dilute solution. Another limiting case is an ideal gas mixture. Here, there are no intermolecular interactions, and the chemical potential of each species has the form:

(23) $$\mu_{\alpha }=\mu_{\alpha }^{*}+kT\ln\rho_{\alpha }\Lambda_{\alpha }^{3}=kT\ln\Lambda_{\alpha }^{3}q_{\alpha }^{-1}+kT\ln\rho_{\alpha }=\mu_{\alpha }^{0ig}+kT\ln\rho_{\alpha }$$

The quantity $\mu_{\alpha }^{0ig}$ is the ideal gas standard CP.

In an ideal gas phase, the pseudo-chemical potential only contains the internal partition function of the molecule of species α, i.e.,

(24) $$\mu_{\alpha }^{*ig}=kT\ln q_{\alpha }^{-1}$$

In most of the discussion of solvation thermodynamics, we assume that the internal partition function of a single molecule ($q_{\alpha }$) is independent of the surrounding molecules. Normally, the interactions between the newly added particle and the other particles in the system might change the energy levels of the molecules and, hence, also the internal partition function. 

We now define the *process* of *solvation* as the process of transferring a particle of species α from a fixed position in an ideal gas phase (or in vacuum) to a fixed position in a solvent. The solvent may be characterized either by the variables $T,V,N_{1},\ldots ,N_{c}$ or by the variable $T,P,N_{1},\ldots ,N_{c}$.

**Figure 2:**
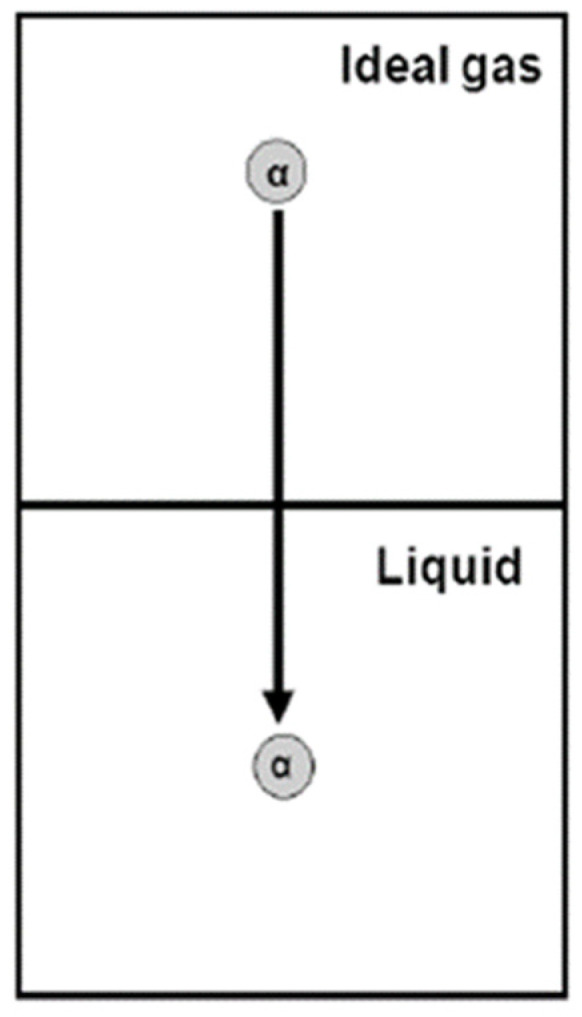
Definition of the solvation process: a molecule *α* is transferred from a fixed position in an ideal gas phase to a fixed position in a liquid phase.

Since the internal partition function $q_{\alpha }$ is presumed to be independent of the thermodynamic variables (either T,V,N or T,P,N), we do not have to specify the condition under which we inserted the particle into the ideal gas. In the solution, we insert the new particle of species α at a fixed position $R_{0}$, either at a fixed T,V,N or T,P,N, Figure 2. 

For simple solutes, we assume that the internal partition function $q_{\alpha }$ is unchanged in the process of solvation. Therefore, the Helmholtz or Gibbs energy of solvation is given by:

(25) $${∆\mu }_{\alpha }^{*}=\mu_{\alpha }^{*l}-\mu_{\alpha }^{*ig}=-kT\ln\langle \exp\left[-{\beta B}_{\alpha }\right]\rangle$$

For a dilute solution of α in the solvent, the quantity ${∆\mu }_{\alpha }^{*}$ becomes identical to the standard “free energy” of solvation for the ρ-process, i.e.,

(26) $${∆\mu }_{\alpha }^{*}\to {\Delta G}_{\alpha }^{0}\left(\rho -process\right)$$

Note, however, that although numerically, ${∆\mu }_{\alpha }^{*}$ becomes identical to ${\Delta G}_{\alpha }^{0}\left(\rho -process\right)$, these two quantities pertain to two *different* processes. This fact brought about the confusion between the solvation free energy (Helmholtz or Gibbs energy) ${∆\mu }_{\alpha }^{*}$ and the *standard* solvation free energy. In fact, some authors erroneously referred to ${∆\mu }_{\alpha }^{*}$ as the “Ben-Naim” standard for free energy transfer based on the ρ-process.

Nowadays, almost everyone who studies solvation quantities uses the process of solvation defined above. Most people trace back the origin of the definition to either reference [7] or Ben-Naim’s (1987) book [1]. In the next section, we will briefly discuss the advantages of the new measure of solvation thermodynamics over the conventional ones. This was discussed to a great detail in reference [1]. 

## 3. The Advantages of the New Definition of the Solvation Process

Since about 1980, one has safely been able to say that almost everyone who studies solvation uses the new definition of the solvation process and the corresponding Gibbs energy of solvation, denoted ${∆\mu }_{\alpha }^{*}.$ Almost no one now uses the old—and now obsolete—standard quantities of solvation. Interestingly, and quite curiously, a few people use exactly the same quantity defined in Section 1 but do not refer to it as “*solvation*” but, rather, as “*excess function*”. This is obviously an inappropriate term to use. The term “*excess function*” is used to describe *deviations* from ideal solutions. There are, in fact, three types of ideal solutions, which are discussed in Ben-Naim (2006) [9]. In fact, the term “*excess function*” was *never* used in the study of solvation phenomena. Only recently, after the new solvation process, as defined in this section, introduced in the late 1970s, that the term “*excess function*” appeared in the literature in the sense of “*solvation process*”.

In this section, we will briefly discuss the advantages of the new measure of solvation thermodynamics over the conventional standard quantities. These were discussed to a great detail in Ben-Naim (1987) [1]. Today, most people working in the field recognize the advantages of the new definition. Therefore, there is no need to discuss those advantages in any detail.

The first “advantage” is actually more a convenience than an advantage. Instead of having to choose between at least three different measures, one has only one. In the old literature, it was not uncommon to find a whole article, presenting tables of data on, say, the “Solvation entropy” of argon in different solvents. Without specifying the choice of a standard state, such data were meaningless. It is like reporting that the temperature in New York is 30, without specifying the units of the temperature. In fact, the situation is even worse; it is like saying the temperature is 30, without specifying the system or the place.

Likewise, saying that the “entropy of solvation” is 100 cal/mol deg, without specifying the process, is meaningless. Once the new measure of the solvation process is universally accepted, then one does not need to repeat the specification of the process.

The second advantage of the new definition of the solvation process follows from the motivation for seeking a new process of solvation. It is that this process and all the corresponding thermodynamic quantities are truly measuring the solvent effect on the thermodynamic behavior of a single molecule in the solvent. In the conventional approach, where at least three “standard” quantities were in use, there was no way to decide, within thermodynamics, which was the best quantity to use. In fact, as was shown in Ben-Naim (1987), one of the standard quantities, based on the mole fraction concentration scale, actually diverges to −∞ when the solvent density tends to zero. Thus, that quantity could not, in principle, serve as a measure of the effect of interactions on the solute molecules.

The third important advantage is that the process of solvation along with all the thermodynamic quantities may be applied to any solvent with any concentration of the solute α. Recall that all the conventional solvation quantities were defined only for dilute solution of α in the solvent. This means that for any pair of a solute and a solvent, there was only one Gibbs energy of solvation. In fact, the very term “solvation thermodynamics” implies the *existence* of a solute and a solvent, and that the solute, say argon, is very diluted in the solvent, say water. In the new process of solvation, no such distinction is necessary. The new quantities may be applied to an infinite range of concentrations of one molecule in another solvent. The latter can be even to a pure liquid, i.e., when there are no solutes or solvents. This led to the study of “self-solvation”, i.e., the solvation of a molecule in its own liquid, a study that could never be carried out with the standard quantities of solvation. Clearly, that is a huge increase in the range of applicability for the solvation process.

Finally, and no less important an advantage, which was relatively difficult to understand, is the following: all the thermodynamic quantities associated with the solvation process can be derived from the Gibbs or Helmholtz energy of solvation. For example, the entropy, enthalpy, and the volume of solvation can be obtained from the standard relationships:

(27) $${∆S}_{\alpha }^{*}=-{\left(\frac{{\partial ∆\mu }_{\alpha }^{*}}{\partial T}\right)}_{P}$$

(28) $${∆H}_{\alpha }^{*}={∆\mu }_{\alpha }^{*}+T{∆S}_{\alpha }^{*}$$

(29) $${∆V}_{\alpha }^{*}=-{\left(\frac{{\partial ∆\mu }_{\alpha }^{*}}{\partial T}\right)}_{T}$$

Thus, ${∆S}_{\alpha }^{*}$, ${∆H}_{\alpha }^{*}$, and ${∆V}_{\alpha }^{*}$ are the entropy, enthalpy, and the volume changes for the *same process* of solvation, as defined in the previous section. Such simple and straightforward relationships for the entropy, enthalpy, and volume could not be obtained for the solvation quantities based on “standard states”. This is discussed in detail in Ben-Naim (1987) [1].

The last advantage is quite important but was not realized by solution chemists until the late 1970s. As noted previously, the Gibbs energy of *solvation* is numerically identical to the Gibbs energy of the process based on the molarity scale.

As an example, the entropy of solvation is given by the derivative in Equation (27) above. However, the entropy change for the standard process, based on molarity, is not obtained by the derivative ${\left(\frac{{\partial ∆G}_{\alpha }^{*}}{\partial T}\right)}_{T}$. Instead, one can show that ${∆S}_{\alpha }^{0}$ is:

(30) $${∆S}_{\alpha }^{0}\left(based\;on\;the\;\rho -process\right)={∆S}_{\alpha }^{*}+kT\;\alpha_{p}^{l}-k$$

Here, *k* is the Boltzmann constant (or the gas constant if we are within the framework of thermodynamics), and $\alpha_{p}^{l}$ is the thermal expansion coefficient of the liquid phase, defined by:

(31) $$\alpha_{p}^{l}=\frac{1}{V^{l}}{\left(\frac{{\partial V}^{l}}{\partial T}\right)}_{P}$$

Similar comments apply to all the other thermodynamic quantities, such as the enthalpy, volume, compressibility, etc. For details on this, see Ben-Naim (1987, 2006) [1,9].

Since the 1990s, there has been a great amount of experimental as well as simulated works on solvation and a few theoretical ones [23,24]. We hope to review all these developments in a future paper.

## 4. Some Applications of the New Measure of Solvation Gibbs Energy

The first, and perhaps the most important, application of the new definition of the solvation process was in the study of *hydrophobic interactions*, or, more generally, the study of solvent-induced interactions (SIIs). Ever since Kauzmann (1959) [6] suggested that the hydrophobic interactions (originally referred to as hydrophobic “bond”) are one of the most important factors in maintaining the stability of proteins, there had been several suggestions as to how to estimate the strength of those interpretations. Fortunately, with the new definition of the solvation process, it was demonstrated that the solvent-induced part of the interaction may be expressed as the *difference* in solvation Gibbs energies of a pair of two solute molecules in water at two distances. More specifically, the potential of mean force (PMF) between two non-polar molecules, say methane molecules in water, may be written as:

(32) $$W\left(R\right)=U\left(R\right)+\delta G\left(R\right)$$

where $U\left(R\right)$ is the direct interaction between the two methane molecules in vacuum, and $\delta G\left(R\right)$ is the SII between these two solute molecules at distance *R*. It is easy to show, based on the definition of the PMF, that $\delta G\left(R\right)$ is related to the difference in solvation Gibbs energies:

(33) $$\delta G\left(R\right)={\Delta G}^{*}\left(R_{12}=R\right)-{\Delta G}^{*}\left(R_{12}=\infty \right)$$

where ${\Delta G}^{*}\left(R_{12}=R\right)$ is the solvation Gibbs energy of the pair of solute molecules at a distance $R_{12}=R$.

**Figure 3:**
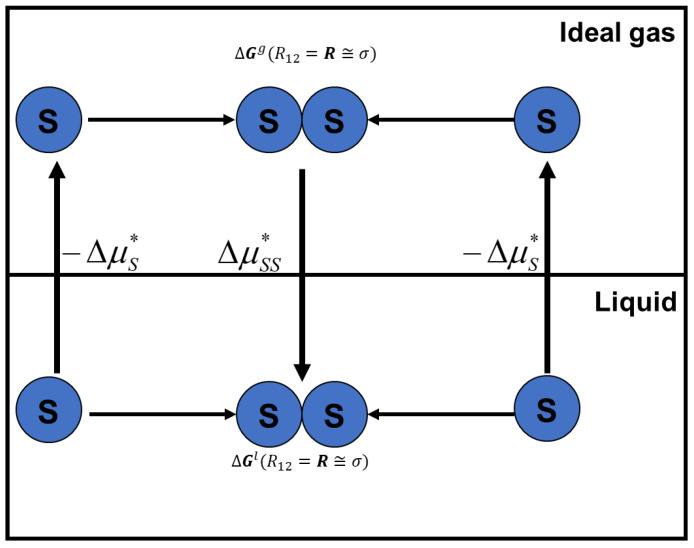
The process of bringing the two solute particles (*S*) from infinite separation $\left(R_{12}=\infty \right)$ to some finite distance ${(R}_{12}=R).$ The process is carried out in an ideal gas and in a liquid phase.

This relationship can be easily “derived” from Figure 3. We conduct the process of bringing the two solute particles (*S*) from infinite separation $\left(R_{12}=\infty \right)$ to some finite distance $R_{12}=R$, then take the difference in the solvation Gibbs energies for the same process in the liquid and in an ideal gas phase, and we obtain:

(34) $${\Delta G}^{l}\left(\infty \to R\right)-{\Delta G}^{ig}\left(\infty \to R\right)=\delta G\left(\infty \to R\right)={\Delta G}^{*}\left(R_{12}=R\right)-{\Delta G}^{*}\left(R_{12}=\infty \right)$$

From this *exact* relationship, one could easily construct an *approximate* measure of the strength of the hydrophobic interaction. As an example, we start with two methane molecules at fixed possibilities but at infinite separation in the liquid. Then, we bring the two solute molecules to a specific distance $R_{12}=\sigma$, where σ is the carbon–carbon distance in ethane. The approximate measure of the hydrophobic interaction for this process is:

(35) $$\delta G\left(\infty \to \sigma \right)≅{∆G}_{Ethane}^{*}-2{∆G}_{Methane}^{*}$$

where, on the right-hand side of Equation (35), we have two measurable quantities, i.e., the solvation Gibbs energies of methane and ethane in water. This measure was used extensively to establish that hydrophobic interactions are indeed unique to liquid water.

Later, in the 1990s, it was realized that what is important in protein folding is not the SII between two non-polar *molecules* (such as methane) but rather the SII between two non-polar groups attached to the protein (such as methyl or isopropyl). This led to the study of the *conditional* solvation Gibbs energy, i.e., the SII between two non-polar groups attached to a backbone. Again, the new definition of the solvation process was useful in the study of the *conditional* hydrophobic interactions.

**Figure 4:**
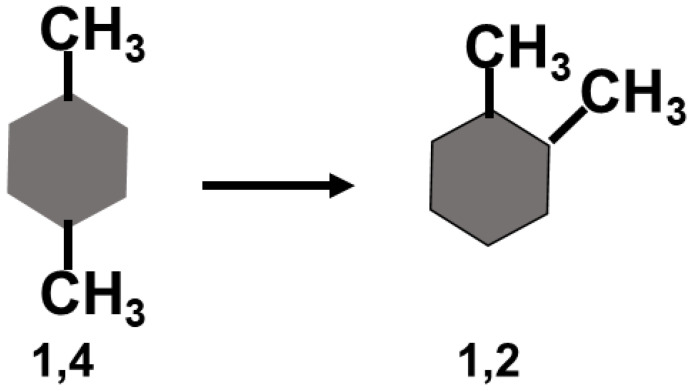
The process of transferring a methyl group from position 4 to position 2, relative to a methyl group at position 1.

As an example, we show, in Figure 4, the process of transferring a methyl group from position 4 to position 2, relative to a methyl group attached to a benzene ring at position 1. The *conditional* hydrophobic interaction between the two methyl groups of positions 1,2 is given by:

(36) $$\delta G\left[\left(1,4\right)\to \left(1,2\right)\right]={∆G}_{1,2}^{*}-{∆G}_{1,4}^{*}$$

On the right-hand side of Equation (36), we have the solvation Gibbs energies of the two isomers shown in Figure 4. Thus, from these experimental quantities, one can estimate the conditional SII between two methyl groups attached to the backbone of a benzene ring.

Before we describe the next revolution in the field of protein folding, we show how solvation Gibbs energies determine the change in a chemical equilibrium constant. Consider the isomerization reaction, which we write as:

(37) U⇄F

The ratio of the equilibrium constants in the liquid and in an ideal gas phase is given by:

(38) $$\frac{K^{l}}{K^{ig}}=\exp\left[-\left({∆G}_{F}^{*}-{∆G}_{U}^{*}\right)/RT\right]$$

where ${∆G}_{F}^{*}$ and ${∆G}_{U}^{*}$ are the solvation Gibbs energies of the F and U isomers, *R* is the gas constant, and *T* the absolute temperature. Comparing Equation (36) with Equation (38), we find that the ratio of the equilibrium constants is determined by the solvent-induced effect on the process of the isomerization reaction in Equation (37).

A particular case of an isomerization reaction is the protein-folding process. In this case, the unfolded protein denoted by U is transformed into the folded form F.

The study of the difference in the solvation Gibbs energies of the folded and the unfolded form led to a complete inventory of all possible solvent-induced interactions between all the groups (both hydrophobic and hydrophilic) attached to the protein. The surprising result of this study was that all solvent-induced interactions between *hydrophilic* groups were found to be much stronger than the corresponding quantities between hydrophobic groups.

The last finding can be said to have revolutionized our understanding of the solvent-induced contributions of the stability of the protein. Until the early 1990s, it was believed that *hydrophobic* interactions are the most important factor in maintaining the stability of proteins. It is now known that solvent-induced interactions between *hydrophilic* groups are far more important, not only in the process of protein folding but also in other biochemical processes, such as the self-assembly of proteins, molecular recognition, and even the solubility of globular proteins [25].

## 5. Some Concluding Remarks

It has been over 40 years since the new measure of solvation thermodynamics debuted. In one of the conferences on solution thermodynamics, the chairman of the session referred to that measure as a “mini revolution”. Of course, that was not a revolution in science in general, as only a handful of scientists were aware of the new measure and appreciated its advantages.

As we noted in Section 4, the new definition of the solvation processes not only contributed to revising the field of solution chemistry but had major effects on other fields of chemistry and biochemistry. In fact, one can safely say that the “mini revolution” in the field of solvation induced a major revolution in biochemistry, more specifically in the study and understanding the process of protein folding, both the thermodynamics and kinetics of this process (the first is associated with the name of Anfinsen [26], and the second is associated with the name of Levinthal [27,28,29,30]). Details of this revolution were discussed in a monograph by Ben-Naim [25].

As noted in Section 3, nowadays, most scientists who are interested in solution chemistry have accepted and used the new measure of solvation thermodynamics as a natural measure, perhaps not aware of the overwhelming confusion that existed in the field in the late 1970s. Some scientists, who use the new measure, refer to it as Ben-Naim’s standard state based on molar concentrations scale. Clearly, they confuse the new *process* of *solvation* with what we refer to as the ρ-process, or the process of transferring a solute from the gas to the liquid at equal molar concentrations, which we denoted as:

$$\left(\begin{array}{c} g\to l\\ \rho_{s}^{g}=\rho_{s}^{l} \end{array}\right)$$

This statement is the result of a misunderstanding. It is true that for the Gibbs energy of solvation, the solvation Gibbs energy is identical to the standard solvation Gibbs energy based on the molar concentration scale. However, this is not true for any other quantity of solvation, such as entropy, enthalpy, or volume of solvation. The reason is that the new measure of solvation thermodynamics *was not* based on a choice of a concentration scale but on a *new* and *different process* of solvation, which was never used before in solvation thermodynamics.

## Data Availability

No new data were created or analyzed in this study. Data sharing is not applicable to this article.
